# A new method of interface tension measurement of a magnetic fluid drop

**DOI:** 10.1016/j.mex.2020.101152

**Published:** 2020-11-19

**Authors:** C.A. Khokhryakova

**Affiliations:** Institute of Continuous Media Mechanics Ural Branch of RAS, 614013, Russia, Perm, Academic Korolev Str., 1, Russia

**Keywords:** Ferrofluid drop, Surface instability, Interfacial surface tension

## Abstract

A new method for determining the interfacial tension of a magnetic fluid (MF) is proposed on the basis of deformation of a MF drop lying on a liquid substrate and subjected to a vertical uniform magnetic field. The results show that the drop elongates in the direction of the field with an increase of its intensity. As soon as the field strength reaches a certain value, the interface and the free surface of the drop become unstable, which causes the peaks of different height to form. It has been found that the ratio of the corresponding critical values of magnetic field intensity is determined by the ratio of surface tension at the interface to that on the free boundary of the drop. Surface and interfacial tension of liquids used in the experiment were measured with the help of tensiometer by the ring detachment method to verify the experimental data. The presented results on the ferrofluid interface tension measurements can be of interest for the specialists in the field of ferrohydrodynamics.

• The magnetic field causes the drop to elongate till the peak instability.
• The critical values of the field strength respond to the ferrofluid initial magnetic susceptibility.
• The ratio of the critical magnetic field values is determined by the ratio of the interfacial tension.

## Graphical abstract

**Figure:**
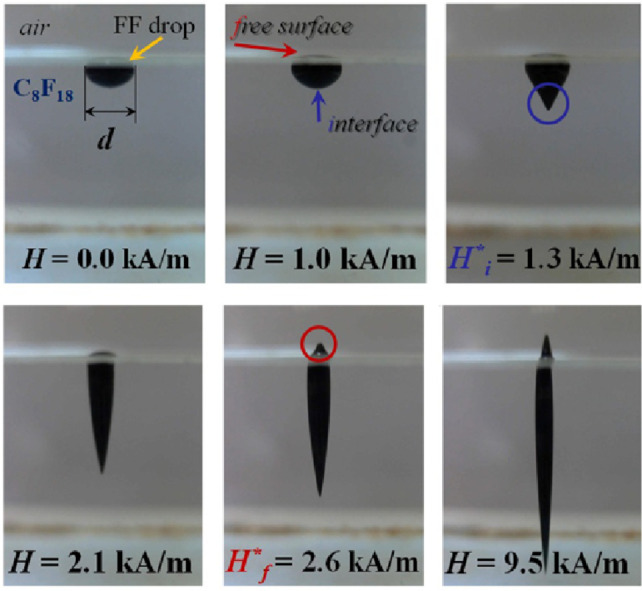

## Specification table

**Table:** 

Subject area:	*Physics and Astronomy*
More specific subject area:	*Thin Films and Interfaces*
Method name:	*Ferrofluid interface tension measurement technique*
Name and reference of original method	*C. Flament, S. Lacis, J. -C. Bacri, A. Cebers, S. Neveu, and R. Perzynski, Measurements of ferrofluid surface tension in confined geometry, Phys. Rev. E, 53 (1996) 4801 [6]*
Resource availability	*NA*

## Background

Direct measurements of surface tension of magnetic fluid being in contact with another nonmagnetic immiscible medium are still under the strong interest of scientists [1,2]. There are two main trends that can be distinguished: the determination of the wavelength of the incipient peak instability of free and interfacial surfaces in an orthogonal magnetic field [3] and the comparison of the MF drop stretched in the longitudinal field with the shape of the modeled drop [4,5]. The first method is limited by the use of a demagnetizing factor of a thin ferrofluid layer, while the second one is determined by the shape of an ellipse or a sphere. The pros and cons of both methods in 2D problem formulation were obtained in [6] where the capillary effects play an essential role due to a thickness of MF located in a Hele-Shaw cell.

In this paper, the object of consideration (floating MF drop) changes its form from an almost flat free surface on the border with the air on one side, and the semi-ellipse at the interface on the other side to the peak instability at both sides. The proposed method is based on the ratio of critical field intensities, so the surface tension could be determined regardless the demagnetizing factor. Another distinguishing feature of this research is the use of direct measurements of the MF surface and interfacial tension with the help of certified tensiometer (Sigma 701), in order to verify the proposed method.

## Materials and equipment

In the experiment, three samples of kerosene-base ferromagnetic fluids, hereinafter called FF 1, FF 2 and FF 3, of equal density (ρ = 1.38±0.02 g/cm³) were used. These samples are also characterized by: different initial magnetic susceptibility χ_0_ and saturation magnetization *M*_S,_ due to the particle sizes *d* of the solid magnetic phase, the phase's volume concentration φ and the average magnetic moment <*m*> of the particles [7] (see Table 1).

**Table 1:** Ferrofluid magnetic properties.

Ferrofluid	χ_0_	*M*_S,_ kA/m	*d*, nm	ϕ	<*m*>, 10^−19^ A m^2^
FF 1	16.5	63	9.6	0.353	3.70
FF 2	2.0	40	7.5	0.362	1.44
FF 3	7.2	50	9.8	0.358	3.05

**Fig. 1:**
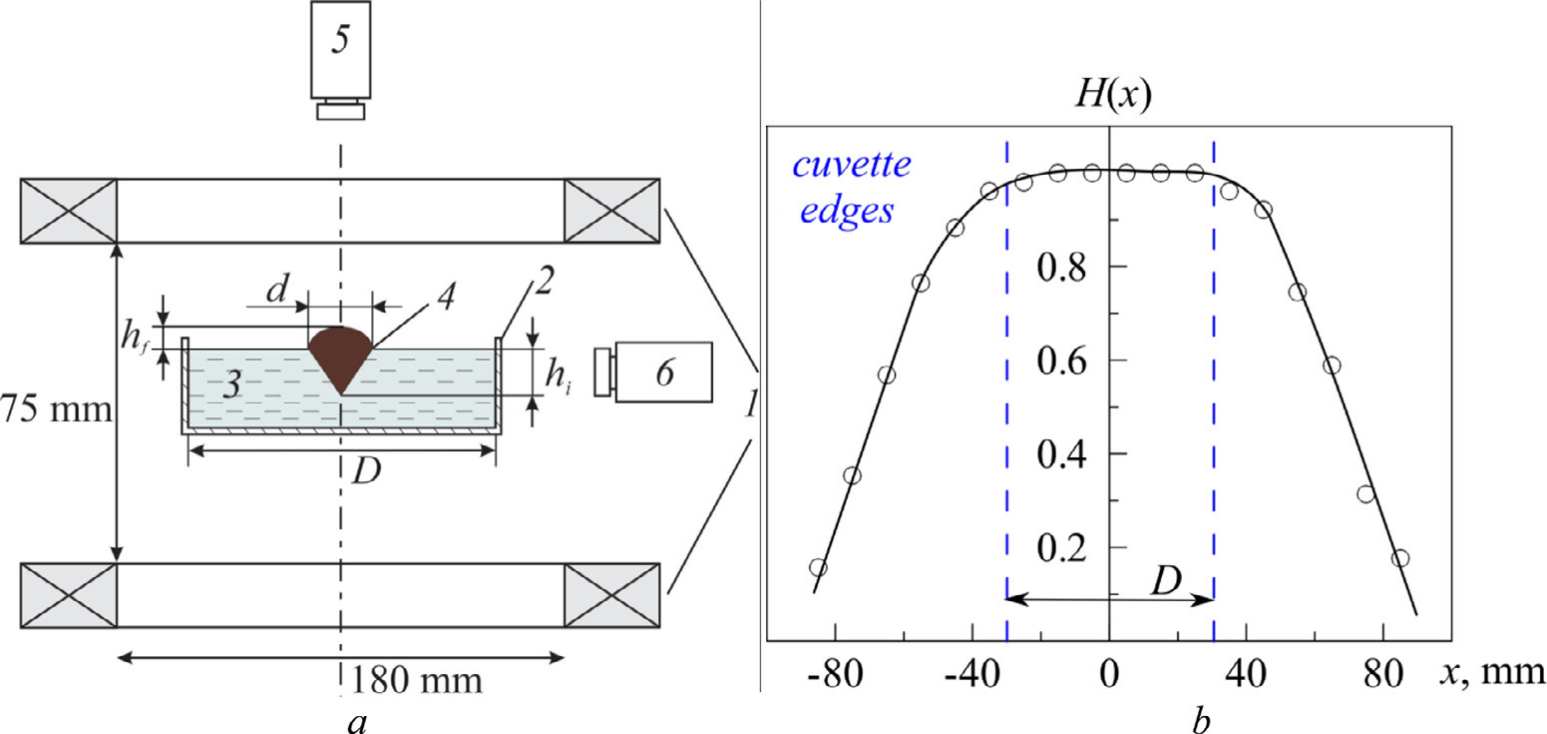
(*a*) Experimental setup: *1* – Helmholtz coils, *2* – square cuvette, *3* – liquid substrate (perfluorooctane), *4* – ferrofluid drop, *5, 6* – cameras. (*b*) The relative (dimensionless) distribution of the field intensity of Helmholtz coils along their diameter in the plane of the cuvette obtained with the Hall Effect sensor.

A glass cuvette with a square cross section 59 mm on a side and 45 mm in depth (Fig. 1) was used as a working cavity. The cuvette (*2*) was placed on a horizontal thin platform between two Helmholtz coils of diameter 180 mm (*1*). The cuvette axis coincides with the axis of the coils. The cuvette was filled with perfluorooctane C_8_F_18_ (*3*) which served as a liquid substrate (ρ_0_ = 1.76 g/cm³) for a ferrofluid drop. The VESTA BM2202 electronic weighing scales were used to determine (with an accuracy of 0.01 g) the substrate mass and, accordingly, its thickness. To this end, a syringe with perfluorooctane was weighed before and after injection of its content into the cuvette. The depth of perfluorooctane substrate was fixed at 40 mm to prevent the FF drop interacting with the boarders. In the absence of a magnetic field the drop used to tend to the side wall of the vessel due to the gradient in the surface tension.

The ferrofluid drop (*4*) was placed on the substrate surface with a mechanical Biohit Proline pipette, which made it possible to determine the drop volume in the range of 5 to 50 μl; the accuracy was 0.1 μl. The volume of large-size drops generated by the syringe was determined by weighing them. The initial diameter of the drop suitable for the method, which, according to calculations, was $d_{0}=\sqrt[{3}]{6V_{0}/\pi }$, varied from 2.5 to 8 mm. The diameters of the drops were obtained within the fixed error not more than 5%.

**Fig. 2:**
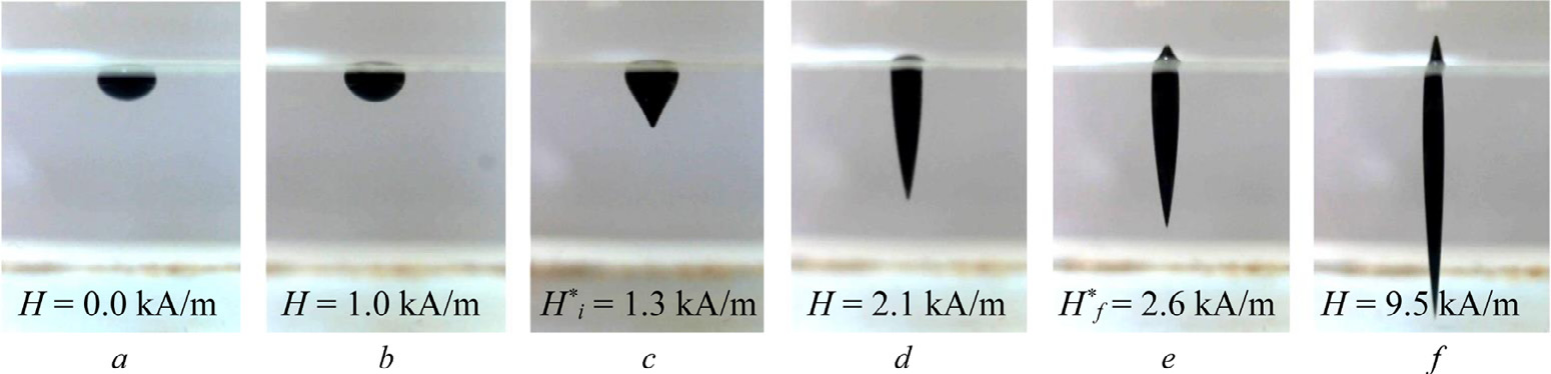
Deformation of FF 3 drop with the growth of magnetic field intensity *H*, kA/m: 0.0 (*a*), 1.0 (*b*), 1.3 (*c*), 2.1 (*d*), 2.6 (*e*) and 9.5 (*f*); *d*_0_ = 2.24 mm.

The strength of the magnetic field *H* generated by the coils was controlled by means of a stabilized power source GPR–7550 D. The magnetic field intensity distribution along the diameter of coils is shown in Fig. 2, *b*. The relative field inhomogeneity obtained by dimensionalizing the measured magnetic field *H* at a given point divided by the value of *H*_max_ in the center of coils did not exceed 1% in both parallel and orthogonal directions. During the experiment, the intensity of the coil current was increased gradually, in small steps, so that each value of the current intensity was held constant for some time to provide a quasistationary shape of the drop. The drop configuration was registered with two video cameras located above the cuvette and on its lateral wall (*5*, 6). All the experiments were carried out at an ambient temperature of (26 ± 1)°C.

## Method

Fig. 2 presents six photographs of the shape evolution of a ferrofluid drop under a vertical uniform magnetic field. In the absence of a magnetic field, the drop takes the shape of two vertical axisymmetrical semi-ellipses of different height conjugated at the interface between the liquid substrate and air, so that the larger semi-ellipsoid is immersed into the liquid, while the smaller semi-ellipsoid comes into contact with air (Fig. 2, *a)*.

A gradual increase in the strength of the magnetic field causes the lower part of the drop to elongate until the field intensity reaches the critical value $H_{i}^{*}$ (Fig. 2, *b*), which is accompanied by the appearance of a peak at the liquid-liquid interface (Fig. 2, *c*). Note that the curvature of the surface changes abruptly and this is indicative of a jump-like pressure redistribution. It should be mentioned, that the peak formation on the FF 2 drops’ surfaces appeared to be a time consuming process due to its low magnetic susceptibility. The critical values of magnetic field for FF 2 were defined for the moment of reforming of peak lateral surface from convex to concave.

When the magnetic field strength increases further, the height of the peak also increases and the immersed part of the drop takes the form of a cone, which elongates along the direction of the field (Fig. 2, *d*). At the next critical value of the field strength $H_{f}^{*}$ the free surface of the drop also becomes unstable, which manifests itself in the formation of the upward-directed peak of the ferrofluid (Fig. 2, *e–f*). As the field strength decreases, the drop shape changes in the reverse sequence, although the disappearance of the peaks is observed at lower values of the magnetic field intensity.

**Fig. 3:**
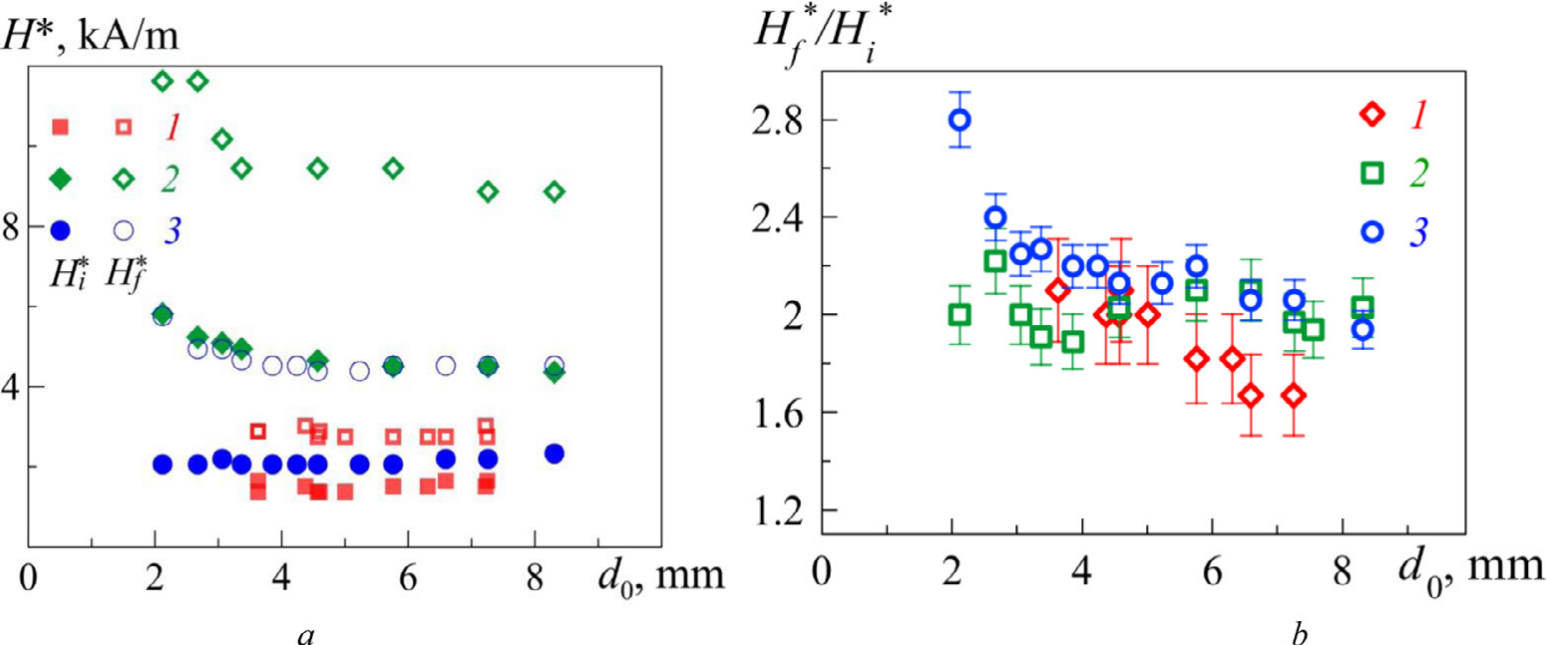
(*a*) Critical value of magnetic field intensity H_i_* (filled symbols), H_f_* (empty symbols) and (*b*) its ratio H_f_*/H_i_* *versus* ferrofluid drop diameter *d*_0_. Magnetic fluids used in the experiment: FF 1 (*1*), FF 2 (*2*), FF 3 (*3*).

The critical values of the field strength H_i_* and H_f_* weakly depend on the initial drop diameter *d*_0_ but strongly respond to the variation of the ferrofluid initial magnetic susceptibility (Fig. 3, *a*). Moreover, H_i_* and H_f_* remain unchanged with decrease in the initial drop diameter to *d_0_* ≤ 3 mm, when the surface forces begin to dominate over the volumetric forces (capillary length λ_c_=(ρg/σ)^1/2^ for the examined ferrofluids bordering the air medium is about 1.5 mm). With an account of the latter restriction, it may be empirically obtained that

(1) $$\frac{H_{f}^{*}}{H_{i}^{*}}=\left(2.1\pm 0.2\right).$$

for all ferrofluids with χ_0_ = (2.0÷16.5) (Fig. 3, *b*) according to the Table 1.

## Method verification

Bearing in mind that the only mechanism inhibiting deformation of the drop in the magnetic field is the surface tension and for a single drop the ratio H_f_*/H_i_* is independent of *d*_0_, we may suppose that H_f_*/H_i_* is a power function of the ratio of the surface tension at the interface σ*_i_* to that on the free surface σ*_f_*.

(2) $$\frac{H_{f}^{*}}{H_{i}^{*}}=k{\left(\frac{\sigma_{f}}{\sigma_{i}}\right)}^{n}.$$

To verify this supposition, it is necessary to determine the surface tension at the interface between the drop and the surrounding medium. However, according to [8], for fluids containing colloidal particles with thin surfactant coating, the Antonov rule is unacceptable. Due to the high sensitivity of a tensiometry system to any external impact the surface tension on the free surface σ*_i_* and interface with perfluorooctane σ*_f_* of the examined fluids were measured in the absence of any external magnetic field. The Sigma 701 tensiometry system (previously KSV instruments Ltd, nowadays Biolin Scientific), that gives at least the σ resolution of about 0.001 mN/m, was used. The ring detachment method [9] provided with the help of Sigma 701 was used in this work to get the surface and interfacial tension of liquid systems in order to evaluate approximately the results of method under consideration. Admittedly, it is noted in the Table 2 that the error of critical field method is of 0.1–0.2 mN/m magnitude.

**Table 2:** The surface tension measurement results.

	$\sigma_{f}$, mN/m	$\sigma_{i}$, mN/m
FF 1	22.6 ± 0.2	4.9 ± 0.1
FF 2	25.1 ± 0.2	6.5 ± 0.2
FF 3	24.3 ± 0.2	5.1 ± 0.3
kerosene	24.2 ± 0.2	5.0 ± 0.1
oleic acid	32.5 ± 0.2	7.9 ± 0.1
C_8_F_18_	13.6 ± 0.2	

*Surface tension measurements were carried out at the average temperature of 27 °C.

As mentioned above, along the experiment the ferrofluid density remained unchanged due to addition of extra kerosene (which was different from that kerosene used for their preparation). Since kerosene is composed of carbon chains that typically contain between 8 and 15 carbon atoms per molecule [10], the length of the molecules and, consequently, the surface tension of fluids varies in the range of 24–28 mN/m. Taking into account the results of previous studies [4,5] it was assumed that the examined surface tension of magnetic fluids should be constant within the range of magnetic field values considered in the experiment (which corresponds to the linear part of magnetization curve). The surface tension measurements are presented in Table 2.

The analysis of the tabulated data allows us to conclude that expression (2) reduces to the following empirical equation

(3) $$\frac{H_{f}^{*}}{H_{i}^{*}}=\sqrt{\frac{\sigma_{f}}{\sigma_{i}}}\;.$$

**Fig. 4:**
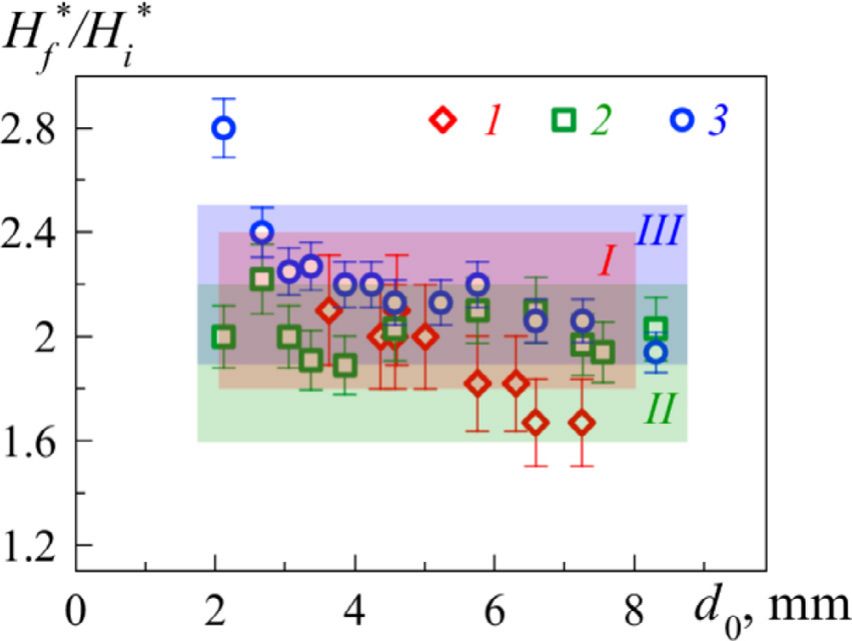
Ratio of critical magnetic field intensity values H_f_*/H_i_* *versus* ferrofluid drop diameter *d*_0_ for the FF1 (dotes *1* and area *I*), FF2 (dotes *2* and area *II*) and FF3 (dotes *3* and area *III*) respectively.

As a result of applying the equation (3), the following data were observed in Table 3. The graphical comparison of the measurements (Fig. 3, *b*) and calculations from the Table 3 are presented in the Fig. 4. Here the highlighted areas *I, II, III* (for FF1, FF2 and FF3 respectively) correspond to the values $\sqrt{\sigma_{f}/\sigma_{i}}$ with respect to confidence intervals.

**Table 3:** Comparison of measurements and calculations.

	$\sqrt{\sigma_{f}/\sigma_{i}}$	H_f_ /H_i_
FF 1	2.1 ± 0.3	2.1 ± 0.1
FF 2	1.9 ± 0.3	2.0 ± 0.1
FF 3	2.2 ± 0.3	2.1 ± 0.1

As follows from the comparison, the experimental value of H_f_*/H_i_* agrees well with the calculation data from formula (3), which opens the possibility of determining the surface tension at the interface between the magnetic fluid avoiding the use of tensiometry system.

The theoretical interpretation of these results could be given according to the following dimensional analysis [6]: the magnetic energy scales as the magnetic field squared, whereas the interfacial energy depends linearly on the surface tension. Along with the energy approach, the problem could be analyzed from the point of the MF drop instability. The classical dispersion equation of surface waves *ω(k)* for ferrofluid [11] is as follows:

$$\rho \omega_{c}^{2}=\sigma k_{c}+\rho gk_{c}-\mu_{0}\chi^{2}k_{c}^{2}H_{c}^{2}.$$

Here the lower index ‘c’ means the critical value corresponding to the instability moment. The critical magnetic field *H_c_* values corresponding to the instability of FF surface are determined by the gravitational-capillary waves on it [12]. Thus, the square dependence between *H_c_* and σ is reasonable*.*

## Conclusion

The conducted experiment revealed a specific character of changes in the shape of a magnetic fluid kerosene-based drop, due to the presence of a liquid substrate of perfluorooctane. The fact that deformation can affect the entire surface of the drop leads to the development of instability at the interface between the drop and the surrounding medium. The following instability causes redistribution of the ferrofluid inside the drop between its upper and lower parts (relative to the interface boundary). It has been shown that the ratio of the critical values of magnetic field intensity is determined solely by the interfacial tension ratio to the free surface tension. This finding may be useful to elaborate non-contact techniques for the measurement of interfacial tension of kerosene-based magnetic fluids.

The surface tension of a magnetic fluid under the action of a magnetic field is the relevant problem that actually could be much wider – the surface and interfacial tension of composite materials in the external fields. This method is surely a trial to resolve this problem. It could help one to get the result for rather low magnetic fields while the surface tension weakly differs from the stationary one. According to [13] the surface tension of magnetic fluid varies in magnetic field depending on its magnitude and direction.

One more significant point is that the form of the object (FF drop) changes during the experiment in a magnetic field that provokes the irregular changes in the demagnetizing factor of the upper and lower borders of the drop. Still, due to the usage of the critical field values’ ratio the demagnetizing factors do not affect the results of the measurements.
